# Methods for numerical simulation of knit based morphable structures: knitmorphs

**DOI:** 10.1038/s41598-022-09422-3

**Published:** 2022-04-22

**Authors:** Sangram K. Rout, Marisa Ravena Bisram, Jian Cao

**Affiliations:** Department of Mechanical Engineering, Northwestern University, Evanston, IL 60208 USA

**Keywords:** Mechanical engineering, Wind energy, Aerospace engineering

## Abstract

Shape morphing behavior has applications in many fields such as soft robotics, actuators and sensors, solar cells, tight packaging, flexible electronics, and biomedicine. The most common approach to achieve shape morphing structures is through shape memory alloys or hydrogels. These two materials undergo differential strains which generate a variety of shapes. In this work, we demonstrate the novel concept that 2D knits comprising of yarns from different materials can be morphed into different three-dimensional shapes thereby forming a bridge between traditional knitting and shape changing structures. This concept is referred to as Knitmorphs. Our computational analysis acts as the proof of concept revealing that knitted patterns of varying materials morph into complex shapes, such as saddle, axisymmetric cup, and a plate with waves when subjected to thermal loads. Two-dimensional circular models of plain and rib developed on CAD packages are imported to the finite element analysis software Abaqus, followed by post-processing into wires and assigning fiber material properties of different thermal coefficients of expansion and stiffness. We also propose potential applications for the concept of programmable knits for developing robots based upon jellyfish like locomotion, and complex structures similar to wind turbine blades. This novel concept is meant to introduce a new field for design when considering morphable structures.

## Introduction

### Shape morphing

The function of a structure is tied to its shape. The benefits of shape morphing involve capturing both features from an undeformed shape to the deformed structure. Shape morphing derestricts the limitations of a fixed shape allowing tailorable structure-based performance on demand^1^. This behavior has been exploited for complex motions, such as microscale locomotion of soft robots, and cargo capture and release^2^, flexible electronics^3^, and in biomedicine to for higher insulin yield and cell viability^4^. In this study we demonstrate computationally that knitted fabrics can deform into complex geometrical shapes under thermal loads. This is made possible by the anisotropic properties of yarn materials^5^ so that strategic spatial arrangement based on the thermal expansion coefficients allow for the design of complex deformations.

**Figure 1:**
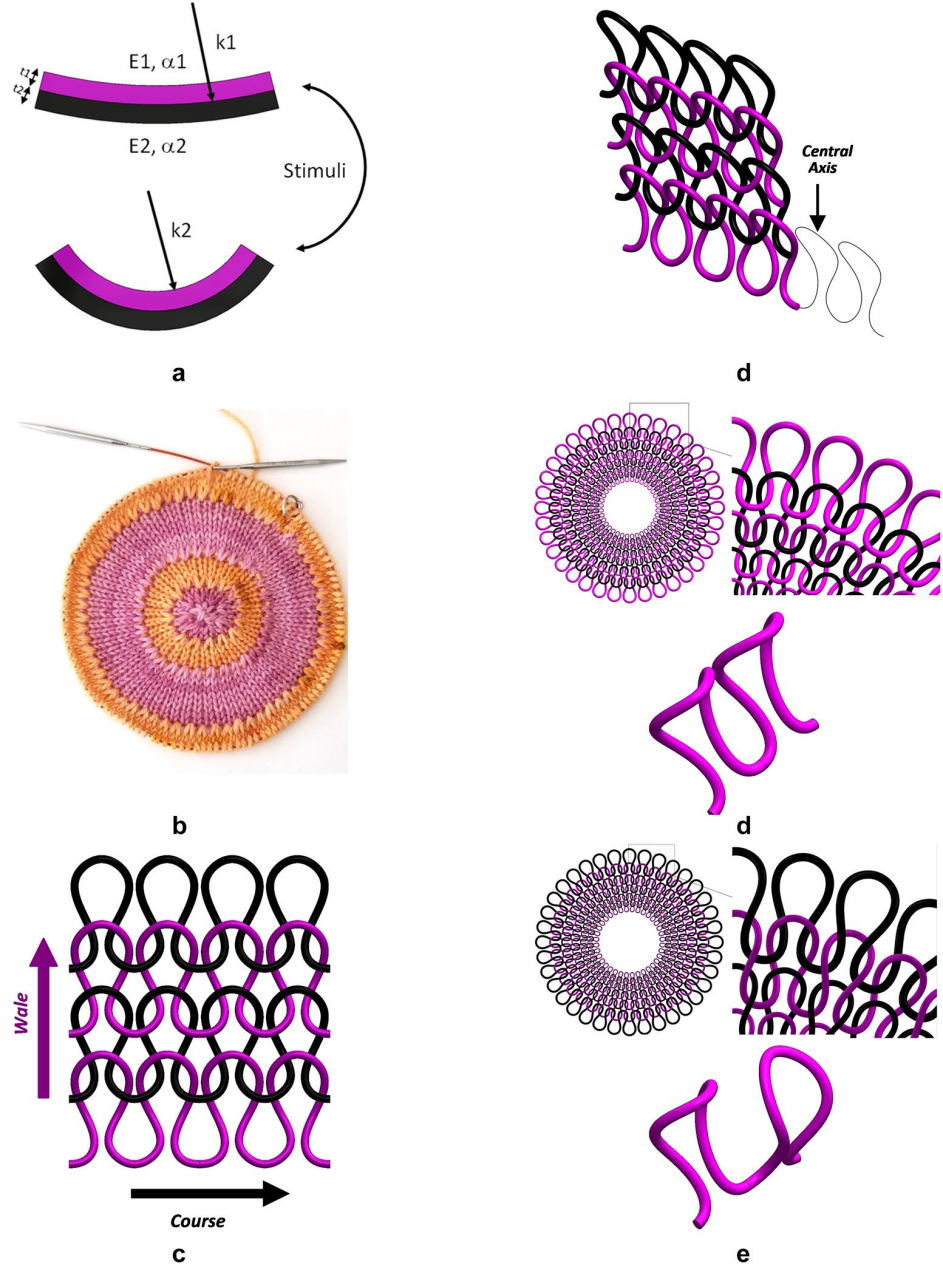
Knitted fabric terminology. (**a**) Bilayer morphing when subjected to stimuli, (**b**) Inspiration for our work. Reprinted with permission. Copyright Staci, (**c**) Schematic showing the wale and course directions of knitted fabrics, (**d**) Central axis of yarn used in Abaqus, (**e**) Plain knit comprises of only knit stitches (or purl stitches) on one side, (**f**) A rib knit consists of alternating rows of knit and purl stitches.

Previous studies in shape morphing have taken the approach comprising of a bilayer structure (Fig. 1a) developed from hydrogels or polymer sheets^5^ that undergo a largescale swelling response with stimuli^6^ followed by studies taking advantage of introducing internal compliance to achieve morphing^7–11^.

Internal compliance variation is achieved through the transition of material between wood polymer composite bilayer and flaps printed from directed thermoplastic polyurethane layer^12^. Other works make use of mechanical architecture such as immobile joints and revolute pairs to achieve complex assemblies upon heating by bending around the joint^13^. Our work accomplishes the end goal of spatial variation of compliance through variation in the yarn architecture; namely the changes in the yarn diameter and geometry which is analogous to internal variations to a disk form shown in previous studies. Moreover, the simulated knitted patterns in this study attain geometrical shapes such as a turbine blade which are relatively more complex than the simple disks or planar shapes limited to soft, thin, sheet-like material that deforms easily^5,14^.

Shape morphing involves changing the curvature of the surface which is defined by the Gauss egregium theorem^15^. According to the theorem, the principal curvatures at any point on a surface can be defined by the curvatures k1 and k2 which lie in orthogonal planes. The product of these curvatures of an initially flat surface is zero and remain unchanged without stretching of the surface in any direction. Simple motions, such as bending or rolling of a paper, lead to change of curvature in only direction, but the intrinsic curvature remains unchanged; a common everyday application is folding of pizza to prevent spillover of the gratings, representing a k1 and k2 product of zero^16^ (see Supplementary Fig. 1). A nonzero product can be represented by activities such as complex surface wrapping by vinyl sheets involves stretching^17^ (see Supplementary Fig. 2).

#### In nature

Differential strain coupled with variation in 3D mechanical architecture that is also found in nature forms the principle of this study. In plants, morphogenesis is primarily driven by differential growth of tissues leading to the formation of complex 3D shapes which take various configurations such as a helical and saddle^18^, the relative growth between the tissues of the gut and attached mesentery drives looping^19^, tendril coiling occurs via asymmetric contraction of the fiber ribbon, the ventral side shrinks longitudinally relative to the dorsal side due to differential lignification affecting the concentration of plant cell hydration, giving the fiber ribbon its intrinsic curvature^20^, and gyrification in the brain arises as a consequence of a simple mechanical instability driven by tangential expansion of the gray matter constrained by the white matter^21^.

#### Engineered shape morphing

The recent focus in morphing structures is expressed through diverse strategies; anisotropic swelling of 3D printed shear induced microfibrils, baromorphs^7^ which use fast pneumatic actuation to transform between negative and positive gaussian curvatures corresponding to the direction of the applied pressure, aeromorph^22^ achieving shape-changing by manipulating the shape of heat-sealed diamond pattern hinges fabricated from various sheets which fold upon pneumatic actuation, millimorphs^23^ which utilized millifluidic chambers, actuated using low boiling liquid for fast actuation schemes at high frequency, thermorphs^24^ which utilize the difference in the residual stress of 3D printed thermoplastic bilayers to achieve morphing when immersed in hot water, hygromorphs^9,25^ which respond to the environmental humidity by changing their shape. Each of these devices rely upon a functionally unique form in actuation and introducing metric to achieve gaussian shapes. Though these engineered forms are enchanting, to fabricate many of these designs requires precision printing or arduous control using photo or magnetic stimuli^2^ or extremely high voltages passing through thin elastomer sheet fabricated layer by layer with carbon nanotube electrodes in between^8,26^; making it difficult for widespread adoption. Here, in this study we present “Knitmorphs”, as class of morphing forms which utilize the fundamental structure of knitting to achieve morphing. This is in contrast to the previous studies in shape morphing fabrics^27^, where they have relied on the anisotropic nature of inflated fabric sheets constrained through seams. Shape morphing behavior has also been demonstrated in the past by the use of materials of different expansion coefficients through the use of bilayers in the work done by Boley^28^. Although the bilayer structured lattices used in their study use printed material which lies in the small strain category in tensile loading, globally they undergo large contraction or growth. The behavior is analogous to the deformation mechanics of knitted fabrics which allow accommodation of large strains due to the inherent compliance in the structure while the individual yarns undergo minimal stretching^29^. Knitmorphs are a relatively simple addition to the shape morphing family that achieve intricate shapes with the added advantage of being porous or a non-continuous surface; thereby opening an avenue for adoption in largescale manufacturing processes.

### Knitted fabrics

#### Mechanics

In recent years several complex problems have taken an interdisciplinary approach which is at the crossroads of the established paths of science and arts, such as origami and kirigami^30^. Knitting- an art form, also viewed by others as an age-old technology could provide a similar unconventional opportunity as origami and kirigami to solve complex engineering challenges^31^. Here in this study, we draw inspiration from a ‘pi knit’ (Fig. 1b) which can be classified as art. Knitted fabrics although lacking in strength in comparison to other fabrics are shown to have high drapability owing to their resistance to buckling and shear deformation^32^. Understanding the physics behind knitted fabrics has been relatively limited in comparison to the mechanics of other manufacturing processes although they have been in use for several hundred years. These limitations are evident from the fact that only recent studies have presented a more accurate model to tracking the units within the fabric structure during deformation^33^ and explanation on laying of yarns^34^.

Tensile tests on knitted fabrics show that stretching follows a J-shape curve wherein a large deformation occurs without much increase in load corresponding to bending of the fiber although there is a variation between the wale and course direction (see Fig. 1c) shown in previous studies^35^. After a certain limit, the fiber jamming occurs which is due to the cross section of the fibers touching against each other leading to increased fabric stiffness^35^. Although knitted fabrics comprise of multiple variables in modeling hierarchy at the structural and material level^36^, in this study we have selected only a few of these variables; namely topology, yarn properties and yarn-yarn interaction into consideration.

#### Shape morphing in fabrics

Earlier studies have exploited knitted architecture by using shape memory alloys to create shape changing behavior such as opening of flowers petals^37^. Moreover, describing knitting as a fourfold hierarchical framework, a variety of shapes were realized using shape memory material with the goal of developing new complex actuators^38^. In this work, we demonstrate that a circular knitted disk comprising of yarns of different material properties looping through adjacent units can morph into various Gaussian curvature profiles. The behavior is in contrast with the individual yarn under the same actuation conditions (as shown in Supplementary Movie 1, 2).

## Material and methods

### Materials

Utilization of materials of different coefficient of thermal expansion being the backbone of the study; the selected materials chosen had relatively high values of thermal coefficient of expansion and contraction along with have low stiffness to facilitate morphing. As the study with basic architecture was done with strain values of + 20% and − 20%, there is a window of opportunity for optimizing other geometrical parameters to use more common materials as demonstrated in other studies^39^.

Twisted and coiled nylon polymers (TCP) fibers can be potential candidates for the experimental work. Their specific work values are shown to be in the order of 2.06 kJ/kg, known to be light by nature yet strong in application, can expand and contract well beyond the 20% strain used in this project^40^ and have relatively low stiffness comparable to the values used in this study^41^. These the desired strain values can be attained at a much lower temperature loading although hard to configure into the knitting dimensions used in this study. From a strength point of view, it has been shown that although these twisted fibers are relatively soft and have low stiffness, when actuated they can support weights of over 50 kg or multiple times its own weight. This shows that there are exceptions to what is typically considered "soft and impractical" materials.

The exact behavior of material is yet to be determined and based upon this understanding the material model should be developed. The constitutive law considered in this model is a linear thermal expansion model

$$\alpha_{L} = \frac{1}{L}\frac{dL}{{dT}}$$

where L is the length measurement and $dL/dT$ is the rate of change of that linear dimension per unit change in temperature.

### Modeling methods

As all of the work presented in this paper is computational in nature, we validate the tensile behavior of knitted structure on FEM with studies done in the past^33^ as shown in Supplementary Fig. 3.

- The method for creation of 3D models for models is discussed below.

Solidworks was used in creating these structures as its graphic interface allows manipulation of the 3D yarn structures, a feature not available in Abaqus. All models prepared in the CAD package were verified to have no interference between the yarns. Since the yarns were modeled as circular beams sections in Abaqus, the imported files had to be post-processed to wire elements which is representative of the central axis of the yarn (shown in Fig. 1d). This was achieved by modeling only a quarter of the yarn (Supplementary Fig. 4) profile in CAD program: Solidworks. All models created in Solidworks were processed in Abaqus using the steps shown in Supplementary Fig. 5 to derive the wire models.

A comparison between the plain and rib knit patterns shown in Fig. 1e,f respectively, shows the plain knit comprises of the knit stitches (or purl stitches) whereas the rib knit has knit and purl stitches alternatively arranged in wale direction. To develop the model of the yarn used in the above configurations, a quarter section of the yarn profile was modeled by passing a natural spline through the loop points. These points were generated by following the methodology shown in Supplementary Fig. 6 using the geometric dimensions shows in Supplementary Table 1 which is used for plain knit pattern. For both plain and knit pattern, a circular pattern of thirty-six sections completed the profile of a single yarn for the plain pattern. For the rib knit pattern, the loop spline was continued with the original yarn mirrored. This pattern of a knit and purl stitch was repeated 18 times to develop into the rib yarn. With boundary conditions specified as a fixed point on the outer yarn followed with application of thermal load from 293K to 696K, morphed shapes of a saddle and an axisymmetric cup were obtained from plain, and rib knit respectively. For the remaining of the geometric figures considered in this paper the same methodology of passing a natural spline through corresponding points has been used for generating the initial geometry and same thermal loads were applied for morphing. The wind turbine blade model was developed by using a linear pattern without the continuity segment between the yarn levels spaced at 2.5 mm for 35 instances. This was followed by creating an instance patterned at 9 degrees around the axis for the second level. The levels were joined by the elbow segment and patterned for 20 instances spaced 18 degrees apart to develop the complete model. Checkerboard pattern was developed by creating a linear pattern of the quarter yarn profile passing through a path defined along the loop points of the smallest unit spaced at 2.5 mm apart for overall distance of 100 mm to build a single yarn. Thereafter 20 instances in the transverse direction, spaced 1.4 mm apart were patterned to develop the complete model. The inverted cone as well as the Plug model has the same yarn geometric layout as the plain knit model. Geometric models for the conceptual work are not described in the paper and can be provided on request from the authors.

- Post processing in Abaqus

On importing into Abaqus through the. STEP format, post processing was done to obtain the wires representing the center of the yarn. The undesired parts were eliminated using the “Geometry edit” toolbar. This quarter section of a single thorough yarn was processed in Abaqus by creating wires from edges followed by removing non-median wire sections (Supplementary Fig. 5). The yarn is simplified to a circular profile. Next, the wire extracted from the earlier step corresponding to the central axis of the yarn is assigned the beam section with material properties and beam orientation along the wire to complete the first yarn. Subsequent fibers in the models were relatively easier to generate. Using the previous yarn as master, subsequent fibers were created as duplicates using “part copy” with a scale factor shown corresponding to the figures. These new yarns were assigned a scaled radius and reassigned material properties shown in corresponding figures. The process was repeated to achieve multiple yarns with different material properties. A thermal loading from 293 to 696 K was applied to the entire model for all the examples illustrated.

The model for plate with waves was developed with a denser knit topology which leads to high volume fraction in the model. For the checkerboard section, alternative positive and negative coefficients material were assigned, spaced at ten knits in the wale and course direction. As an alternative strategy the diverse set of expansion coefficients used in some of the examples can be attained by actuating the fiber made material of fixed thermal coefficients to different degrees of stimuli (or load) as an alternative strategy.

Limitations of the beam element are discussed in Abaqus documentation. Beam element is appropriate for modeling the yarn as it is close to a one-dimensional approximation considering the cross section is small compared to the dimension perpendicular to the axis of the beam. Moreover, as the cross section is considered solid, it is not prone to display softer bending behavior shown by pipe or I-section beams^42^. A refined mesh for first order beam elements captures the initial curvature of the yarn well.

Considering the vast number of contacts involved in the structure and the significance of sliding of yarns contributing to the overall deformation of the knits^29^ the explicit solution method was used in Abaqus as it can handle complex contacts with relative ease. Another limitation in Abaqus (similar with Ansys) is the lack of opportunity on running mesh convergence studies for beam elements. Although, small element sizes lead to converged results, for cases with large number of interactions they facilitate penetration between beams. The approach undertaken in this study composed of a refined mesh, with multi-node elements capturing the curvature of the beam which according to other studies provides accurate results^42^.

Since the minimum stable time increment was small, mass scaling was added to speed up the simulations. To ensure the accelerated analysis is not affected by dynamic forces such as inertia, a comparison of kinetic energy to strain energy was verified to be within a small fraction. Supplementary Fig. 7 demonstrates few models in this paper which are shown to have negligible effect of mass scaling on the analysis. Detailed information can be found in Abaqus documentation^42^.

A dynamic explicit analysis procedure was adopted for the study with time-period of 1.5 s. With mass scaling for the whole model set to 5; a target time increment of 1E-6 was selected to reduce the computation time. The interaction between the wires was modelled as general interaction containing the properties of tangential behavior 0.12 and normal behavior as “hard contact”; adopted from the default interaction between yarns from TexGen^43^. From the outermost yarn, an arbitrary node among the outermost section was fixed to allow the structure less restricted freedom to morph. A thermal loading from 293 to 696 K was applied to the entire model comprising of yarn with different material coefficients. Thermal loading is selected because coefficient of thermal expansion is available for most composite fibers, is simple form of stimulus as it can be achieved multiple methods such radiant heating, joule heating or convective heating. Moreover, it can easily be implemented for prototyping as well as common at industrial scales. Although the thermal loading was consistent in all the analysis; it is not hard-set. The key here is the strain values which can be obtained through any other form of loading or stimulus. Although knits have a varying amount of pretension if done by hand and more consistent if using machine, this study did not consider the pre-tension in the yarn. The addition of pretension can be done through the S11 value in Abaqus modeling.

## Results

### Basic architecture

**Figure 2:**
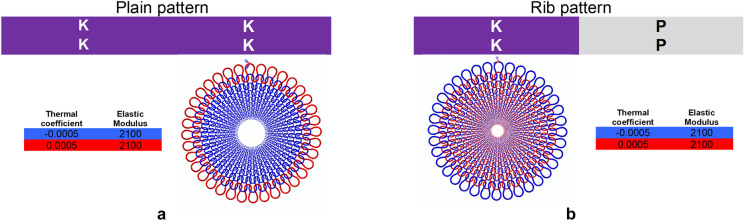
Basic architecture and material. (**a**) Plain knit pattern has only knit stitches (when seen in opp. to plane of figure purl stitches only) adjacent to each other as shown in the schematic on the top. Material properties defined using the color coding with blue representing negative thermal coefficient of expansion while red represents positive thermal coefficient of expansion with insert showing corresponding yarns color. (**b**) Rib knit pattern has knit stitches adjacent to purl stiches as shown in the schematic on the top. Material properties defined using the color coding with blue representing negative thermal coefficient of expansion while red represents positive thermal coefficient of expansion with insert showing corresponding yarns color.

**Figure 3:**
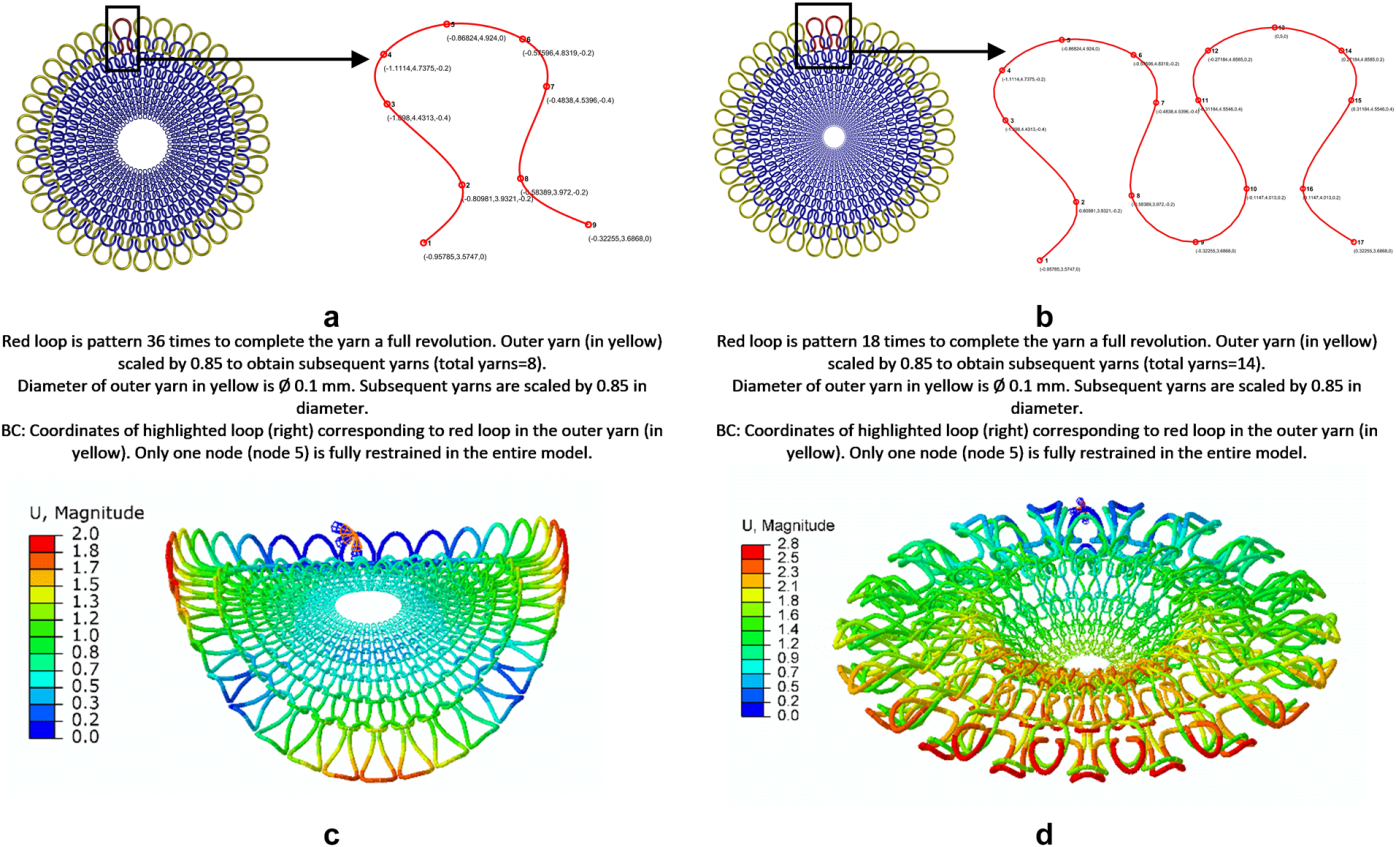
Basic architecture-based morphing. (**a**) Material properties defined use the color coding for the plain and rib knit. (**b**) Blue represents negative thermal coefficient of expansion whereas red represents positive thermal coefficient of expansion. (**c**) Geometric properties of the a single loop highlighted in red, which is then patterned. (**d**) Geometric properties of the a single loop highlighted in red, which is then patterned. (**e**) A plain knit morphs into a concave saddle shape. (**f**) A rib knit morphs into axi-symmetric cup shape.

Commonly used stitches in knitting are the knit stitch and purl stitch, which are canonical in nature; the reverse of knit is purl and vice-versa. Using various combinations of these stitches leads to the fundamental patterns in knitting: plain knit and rib knit as demonstrated. Plain pattern comprises of the same form of stitch with its neighboring domains row-wise (course) and column-wise (wale) whereas rib stitches alternate between knit stitch and purl stitch row-wise (course) and with no change column-wise (wale). The initial setup for the basic architecture consisted of using plain-knit pattern with the material sections defined as color coded (Fig. 2a) and using rib-knit pattern with the material sections defined as color coded (Fig. 2b), with the outermost yarns defined by following the unit loop as per Fig. 3a,b. Application of thermal loads from 293 to 696 K, led to morphed shapes of a saddle shape (Fig. 3c and Supplementary Movie 3) and an axisymmetric shape (Fig. 3d and Supplementary Movie 4) respectively.

**Figure 4:**
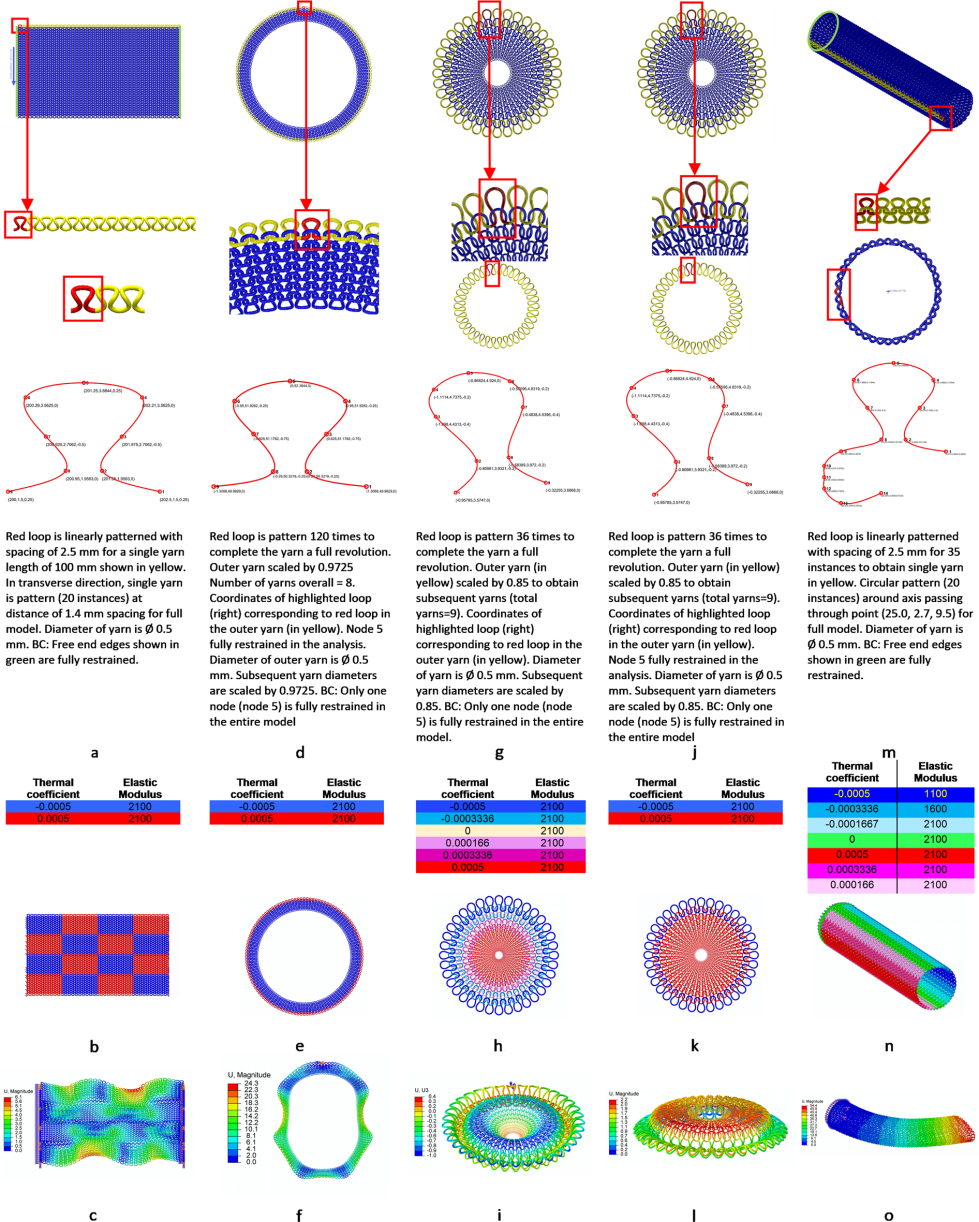
Distortion of various shapes with programmed material –Geometric and Material properties and final morphed structure. (**a**) Initial structure of checkerboard pattern with edges along y constrained. Insert shows a single loop highlighted in red, which is then patterned linearly to complete a single yarn. Linearly repeating yarn is shown in yellow. Geometric coordinates of the smallest repeating unit shown in red for the yarns corresponding to the insert in previous row. (**b**) Material coding for checkerboard pattern. (**c**) Final morphed structure of the checkerboard pattern. (**d**) Initial structure of plate with waves pattern of high-volume fraction. Insert shows a single loop highlighted in red, which is then patterned linearly to complete a single yarn. Radially repeating and scaled yarn is shown in yellow. Geometric coordinates of the smallest repeating unit shown in red for the yarns corresponding to the insert in previous row. (**e**) Material coding for pattern with waves. (**f**) Final morphed structure of plate with waves. (**g**) Initial structure of inverted cone. Insert shows a single loop highlighted in red, which is then patterned linearly to complete a single yarn. Radially repeating and scaled yarn is shown in yellow. Geometric coordinates of the smallest repeating unit shown in red for the yarns corresponding to the insert in previous row. (**h**) Material coding for inverted cone. (**i**) Initial structure of inverted cone. (**j**) Initial structure of plug. Insert shows a single loop highlighted in red, which is then patterned linearly to complete a single yarn. Radially repeating and scaled yarn is shown in yellow. Geometric coordinates of the smallest repeating unit shown in red for the yarns corresponding to the insert in previous row. (**k**) Material coding for plug. (**l**) Final morphed structure of plug. (**m**) Initial structure of wind turbine blade. Insert shows a single loop highlighted in red, which is the patterned linearly. Circumferentially repeating yarn is shown in yellow. Geometric coordinates of the smallest repeating unit shown in red for the yarns corresponding to the insert in previous row. (**n**) Material coding for Wind turbine blade. (**o**) Final morphed structure of the wind turbine.

One explanation for the possible difference in shapes obtained is that the rib pattern is inherently symmetric in nature due to the alternate patterns of knits and purls. For the checkerboard pattern (Fig. 4a), alternative positive and negative material coefficients properties were assigned (Fig. 4b and Supplementary Movie 5) leading to resulting morphed structure of the checkerboard pattern (Fig. 4c). Using a plain pattern with higher volume fraction where Fig. 4d demonstrates the geometric properties of the smallest unit and material properties defined in Fig. 4e provide us with a morphed plate with waves (Fig. 4f for dynamic see Supplementary Movie 6). These results are similar to ones reported in other studies where the number of waves depends upon thickness^14^ although in this study the correlation was not examined. Using materials on the geometrical configuration of plain pattern as Fig. 4g (which identical to Fig. 2a) and material defined in Fig. 4h can lead to different morphed shape- an inverted cone (Fig. 4i, dynamic see Supplementary Movie 7). Again, using materials on the geometrical configuration of plain pattern as Fig. 4j (which identical to Fig. 2a) and material defined in Fig. 4k can lead to raised dome (Fig. 4l, dynamic see Supplementary Movie 8).

### Potential applications

#### Composite manufacturing

The shapes obtained in this study demonstrate the potential to create complex shapes such as oil pan bottom, car fenders, engine casings through this manufacturing technique. In addition, development of prototypes can be accomplished bypassing the need to create complex molds. Just as incremental sheet metal forming^44^ has become a popular manufacturing method for dieless forming technique, morphing fabrics can be the cornerstone for dieless forming of composite suitable for bespoke items and rapid prototyping. This reduction of initial investment in capital equipment and tooling with low cycle time would significantly reduce cost per unit stiffness^45^ to be at par with steels, which has to be bought down to 0.75$/lb-200 GPa from present 4.35$/lb-100 GPa. Moreover, since the process can be fully automated, it makes it suitable for industrial applications. This would bring down the barriers obstructing the widespread adoption of composites in different manufacturing sectors such as the automotive industry.

- Large scale structures

Wind turbine blades, one of the largest composite structures built, are fabricated using extremely complex processes with large molds, which contribute to a third of the cost of the fixed costs^46^. One approach to reduce the cost of such structures could be to use a strategy as shown in our work. Using geometric properties defined in Fig. 4m and by combining different thermal expansion properties and gradients in stiffness Fig. 4n, it is possible to achieve a blade like shape as shown in Fig. 4o and Supplementary Movie 9. The blade section was designed with a circular pattern in the wale direction while in the course direction the profile was linearly patterned. One end of the cylindrical section was assigned fixed boundary conditions similar to the root of a blade.

Moreover, morphing can be harnessed to improve the overall efficiency by adapting the profile to the wind speed. Furthermore, the patterns can be combined to create new forms; the use of a rib knit and plain knit with programmed materials can form the shape of NASA’s LDSD.

#### Robotic and biological systems

**Figure 5:**
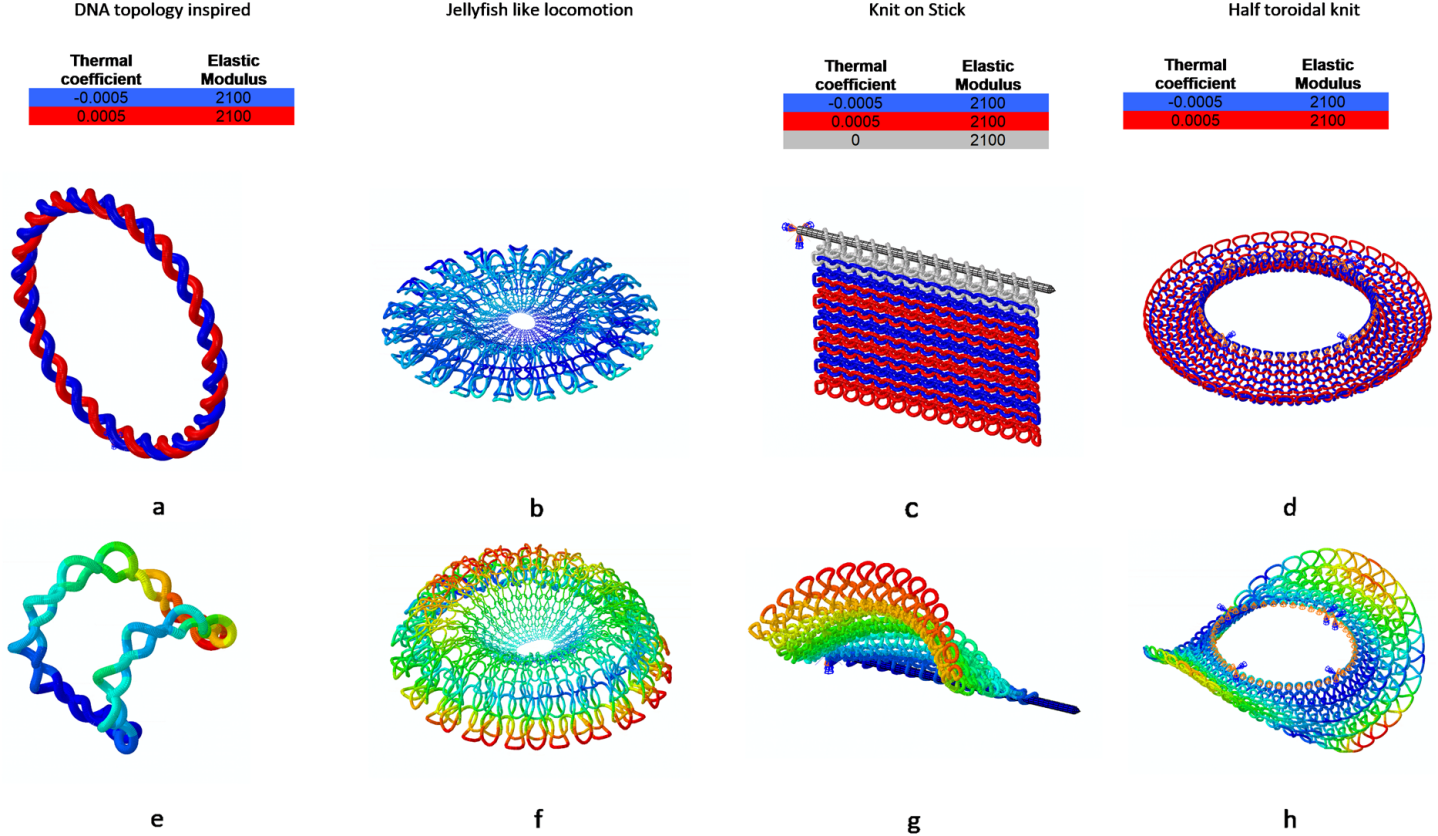
Conceptual work and inspiration. (**a**) DNA coiling inspired topology. Color coded material properties defined. (**b**) Jellyfish like aquatic motion can be achieved by alternating between the two configurations shown. Config 1. (**c**) A multi material knit on knitting needle. Color coded material properties defined. (**d**) Half toroidal composed of multi material yarns. Color coded material properties defined. (**e**) Morphed structures of the coiled DNA. (**f**) Jellyfish like aquatic motion can be achieved by alternating between the two configurations shown. Config 2. (**g**) Morphed structure of multi material knit. (**h**) Morphed structures of the Half toroid.

Inspired by topological changes frequently observed in DNA molecules which undergo continuous morphing to perform function^47^, a similar pattern with different material properties is studied. Figure 5a shows the structure inspired from coiled DNA, with strands made from materials and the resulting morphed structure Fig. 5b. Similar to the ways in which origami has been used at a cellular level, here we drawn inspiration from cellular level to new kinds of knitting architecture.

Other applications include developing a robotic system which mimics the swimming behavior of Jellyfish by alternating between the open and closed forms to carry payload to a site or be deployed as emergency shelters (Fig. 5c,d).

#### Conceptual work

In addition to the above application, a few conceptual works are presented.

Figure 5e,f shows the initial configuration of a knit on wooden needle and final morphed structure on a wooden knitting pin respectively. Figure 5g is a representation diagram from a half-section of a toroid composed of knitted fabric made of different material and the final morphed configuration in Fig. 5h.

## Discussion

Although in this study no experimental validation was successfully performed, due to the challenges of knitting at the scale of these structures by hand and limitation on resources, the significance of this work holds in the opportunities it presents, as part of an early innovation cycle where the technology isn’t fully mature but can foster development by providing a novel approach to morphing.

Scaling the diameter of the yarns and the overall architecture plain knit architecture (a) to factors of 10 and 100 did not yield the same effects of morphing behavior as shown in Supplementary Fig. 8. This observation can be attributed to effects of friction which is non-linear in nature and the fact that the bending stiffness scales by the ratio of the third power of the diameter. The use of material combinations with thermal coefficient of expansion values of ± 5E−6 and ± 5E−5 on the plain knit pattern in a did not yield morphing behavior. Detailed investigation of the appropriate material properties in combination of the geometry which lead to morphing may be conducted in a future study. A parametric study conducted (variables as number of loops, and depth of the plane d1 and d2) did not provide conclusive insight for relationships between the variables and the out of plane behavior. Moreover, our survey determined that although certain fibers exhibit negative coefficient of thermal expansion, the non-linearity along with the low expansion modulus makes them challenging to incorporate^48^. The response of thermal systems is much slower in comparison to pneumatic actuation due to the thermal inertia of the system which makes the implementation into applications such as flight is challenging. Carbon fibers and other typical composite fibers are difficult to knit due to short loop, size of tow in knitting machines and fuzz, thereby requiring a special modification. In knitted structures, it is not possible to reach conventional fiber volume content which lead to low mechanical properties compared to conventional uni-directional composites. Other ideas which may be pursued out of curiosity-based research can be on knot theory, fractals, braiding and defects.

Knitting along with braiding, can be explained by knot theory with the common theme of topology. Knot theory has advantages in allowing one to provide an algorithmic approach to determine structure–property relationship or topological property. With the topological invariants being defined as but not limited to the number of crossing, loop length, maximum and minimum yarn curvature with elimination of duplicates through rules based on Reidemeister moves, provides the freedom for exploring a large number knitting architecture configurations using advanced techniques with the goal to determine the optimal material and architecture combination for desired performance^49,50^.

A fractal is a never-ending pattern, where the pattern at a granular level matches the top levels. Knitting, which can be broken down as a recursive programming with self-replicating patterns, can be extended to a fractal form. Although, there is a mist surrounding whether a possible behavior/observation can be classified as a fractal mathematically, the view of knitting as fractal here is at a lower level, visually represented. Fractal based textiles can be used for engineering advanced textiles at the nano to macro levels for regulation of sweat or be developed into smart fabric-based wearables.

Braided composite fibers are valued for properties such as high impact strength, are typically seen in asymmetrical components such as diffuser sections or circular shaped mandrel. Cases when using non asymmetric cross-section, varying along the length are termed such as complex shapes^51^. Alternative methods to manufacture complex shapes in the past are based on expandable braided sleeves. Here a proposal is made wherein using different material properties from a tubular braided sleeve, morphing can be exploited to realize complex shape non-axisymmetric shapes. Moreover, another proposal would be using braids made from different material as the yarn for knitting.

The effects of imperfections such as holes, dropped stitches, thick or thin yarn, broken needle is likely to alter the morphing behavior similar to any buckling problem and therefore one may ask if it possible to pre-program these defects for positive outcomes. Earlier studies have demonstrated that introduction of topological defects in phase transforming metamaterials, providing interesting results^52^.

The axial strains from the yarns when constrained by the geometrical topology of the knit structure leads to interesting morphing structures which are expressed through bending of the yarns. Furthermore, devising inverse models which can accurately predict the shape after loading would be necessary to develop significant understanding of the process as it has been demonstrated through the work with other manufacturing processes^53^. Extending the work on Lagrangian mechanics to explain the mechanics of knitmorphs by addressing the topological changes within the different architectural combinations would be another interesting research endeavor.

The arrangement of yarns of different material properties in specific order leads to certain meaningful shapes such as saddles, inverted cup, etc. Yarns arranged in any random arrangement do not show the same characteristics. It is a reasonable assumption that state that the results are a combination of material and the structure. Moreover, the design space needs to be explored and an analytical formulation be determined, to gain a better insight to which factors are critical to exercising control over the degree of morphing behavior.

Experimental strategies conceived during this research using extreme thermal expansion values which limits practical material options but seeking non-conventional manufacturing techniques such as 3D printing may hold the key. There is a plethora of architectural possibilities in knitting for research exploration; both documented and undocumented beyond the two common ones (plain and rib) that is explored in this work. The studied knitted patterns although developed through simple architectures have shown a rich behavior and can mature into mainstream research using machine learning techniques.

## Supplementary Information

Supplementary Video 1.

Supplementary Video 2.

Supplementary Video 3.

Supplementary Video 4.

Supplementary Video 5.

Supplementary Video 6.

Supplementary Video 7.

Supplementary Video 8.

Supplementary Video 9.

Supplementary Figures and Table.

## Data Availability

All data and models used in this study can be provided by the authors on reasonable request.
