# Supplementary material for: Methods for numerical simulation of knit based morphable structures: knitmorphs
Source: Sci Rep. 2022 Apr 22;12:6630. doi: 10.1038/s41598-022-09422-3 (PMC9033797; doi:10.1038/s41598-022-09422-3)
Supplement: Supplementary file 10 — Supplementary Figures and Table. [file 41598_2022_9422_MOESM10_ESM.docx]

Supporting Information

Shape morphing of knitted fabrics

Sangram K. Rout * 1, Marisa Ravena Bisram 1, Jian Cao 1,

1 Department of Mechanical Engineering, Northwestern University, Evanston, IL 60208, United States.

*Corresponding author

E-mail address: [sangramrout2021@northwestern.edu](mailto:sangramrout2021@northwestern.edu)

Movies

Movie S 1 Single unit of knit made of compression material under actuation

Movie S 2 Single unit of knit made of expansion material under actuation

Movie S 3 Formation of Concave saddle starting from 2-D Plain knit of different material properties

Movie S 4 Formation of AxiSymmetric Cup starting from 2-D Rib knit of different material properties

Movie S 5 Formation of Distorted checkboard pattern from Plain knit of different material properties

Movie S 6 Formation of Plate with waves starting from a high-volume fraction. 2-D Plain knit of different material properties

Movie S 7 Morphing of Plain knit of different material properties into inverted cone

Movie S 8 Morphing of Plain knit of different material properties into a cup

Movie S 9 Formation of Wind Turbine starting from a configuration as shown in Table S 1 & Supplementary Figure 7


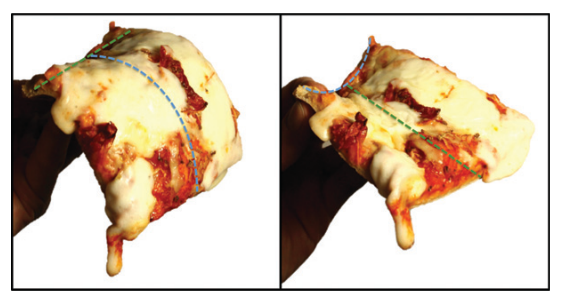


Supplementary Figure 1. Gaussian Curvature allows us to hold pizza correctly. ^1^

|  | 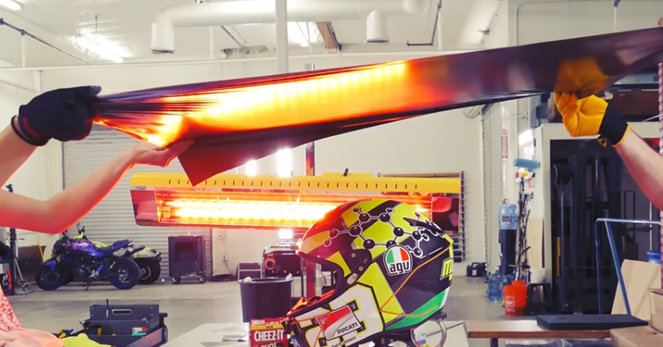 |
| --- | --- |
| a. | b. |
| 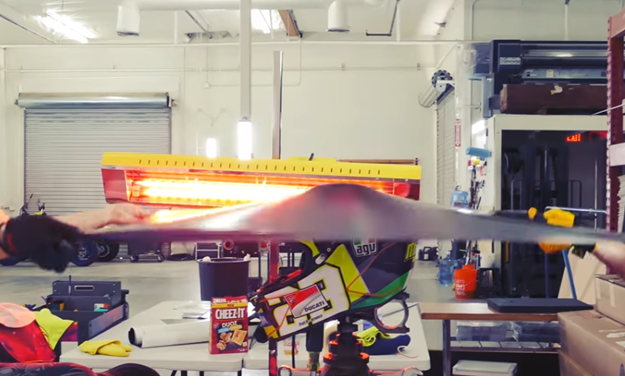 |  |
| c. | d. |

Supplementary Figure 2. Vinyl wrapping of motorcycle helmet. ^2^

a. Preheated flat vinyl wrap

b. positioning flat on the top of helmet

c. Initial stretch

d. Layup and hand pressing to stretch

1. Initial configuration of knit ^3^. It is not necessary to obtain permission to reuse this article or its components as it is available under the terms of the [Creative Commons Attribution 4.0 International](https://creativecommons.org/licenses/by/4.0/) license.
2. Final configuration of knit after stretching ^3^. It is not necessary to obtain permission to reuse this article or its components as it is available under the terms of the [Creative Commons Attribution 4.0 International](https://creativecommons.org/licenses/by/4.0/) license.
3. Stretching behavior as recorded experimentally/Lagrangian theoretical model ^3^. It is not necessary to obtain permission to reuse this article or its components as it is available under the terms of the [Creative Commons Attribution 4.0 International](https://creativecommons.org/licenses/by/4.0/) license.
4. Initial configuration of knit.
5. Final configuration of knit after stretching.
6. Stretching behavior as recorded experimentally/analytical model; (Insert) Mechanical behavior of biological tissue such as artery (Singh et al., 2015))

Note: As seen by using the deformation mechanics, the yarns at the edges tend to curl up in both the experimental as well as FEM model. One possible explanation for this behavior is that the tension in these sections falls considerably in comparison to the other sections ^3^. Note: The dimensions of the unit knit is not to scale, material properties are not the same

Supplementary Figure 3 Validation of FEA with experimental and analytical model


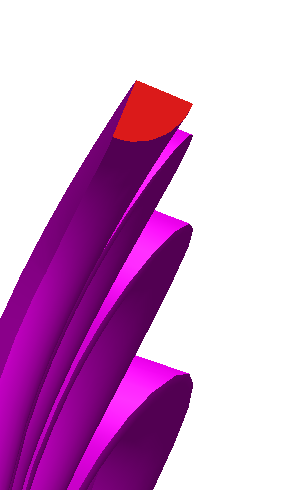


Supplementary Figure 4 Quarter model of the yarn imported into Abaqus for post-processing into wire/beam sections.

a. Importing the CAD model generated in SOLIDWORKS using the. Step exchange format b. Generating set by selecting central axis of wire c. Removing all other wire sets which do not fall on the central axis of the wire d. Resulting central axis of the wire after postprocessing e. Assigning of the wire orientation in tangential orientation of the wire f. Assigning sections to the wire with material defined and circular profile g. Resulting beam profile.

Supplementary Figure 5 Steps involved from importing a quarter model to post processing in Abaqus.

Supplementary Table 1 Geometric properties considered

| Parameter | Description | Values |
| --- | --- | --- |
| θ1 | Side wall angle | 20 ֯ |
| θ2 | Angle subtended at center | 10 ֯ |
| R | Top radius | 0.331 mm |
| r | Bottom radius | 0.243 mm |
| d1 | Dist. plane 1 | 0.2 mm |
| d2 | Dist. plane 2 | 0.4 mm |

Supplementary Figure 6 Geometrical dimensions of outermost yarn used in figure 3

a. Corresponding to Table 1 a Front view of yarn b. Side view of the knitted fabric. c. Central axis of yarn

c.

b.

a..


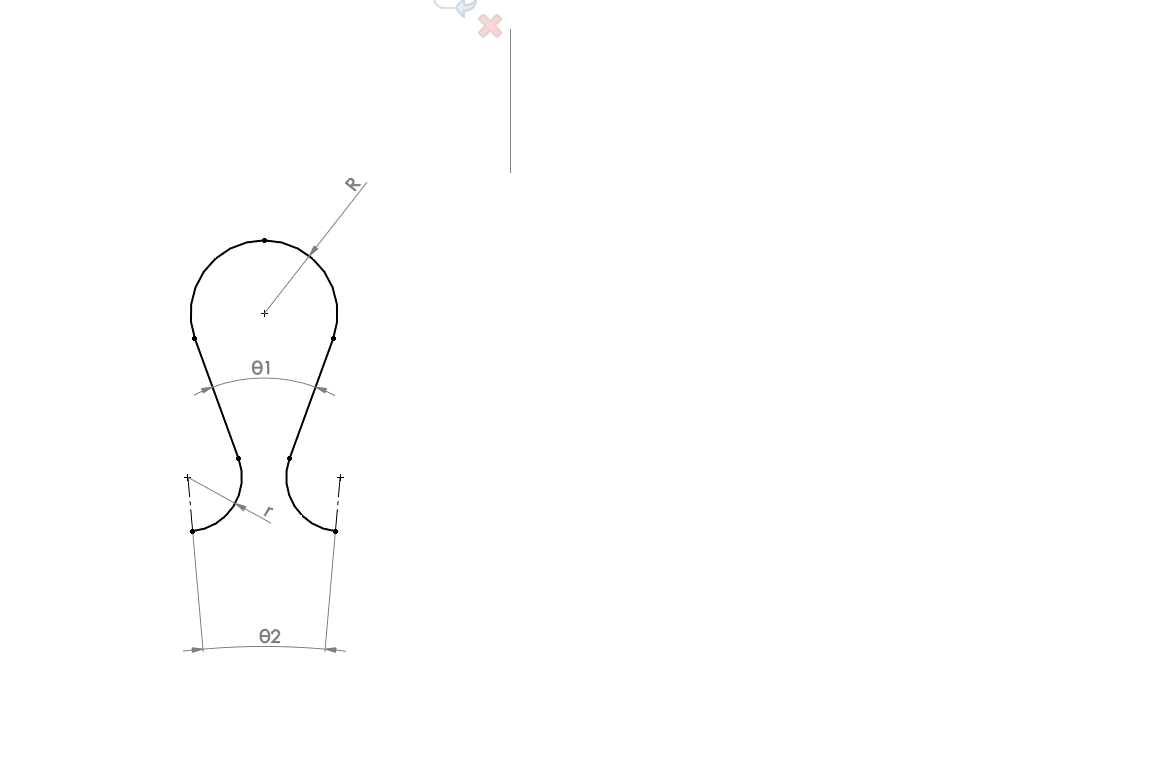

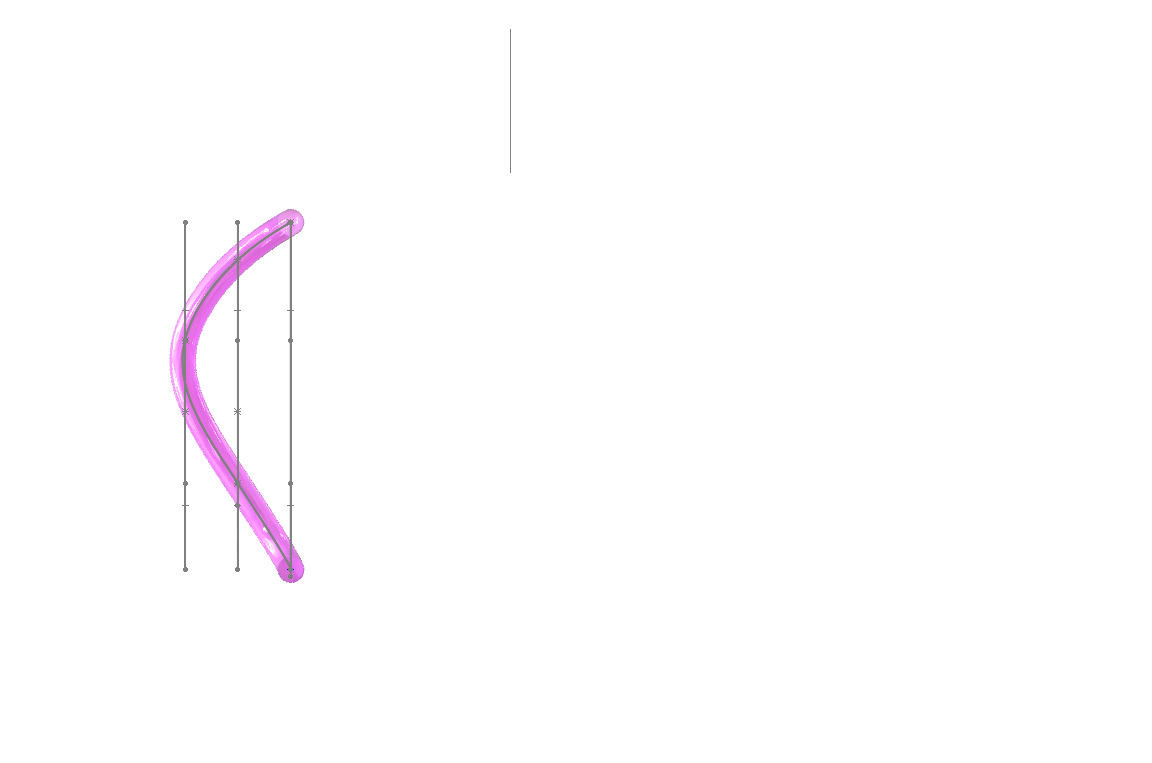

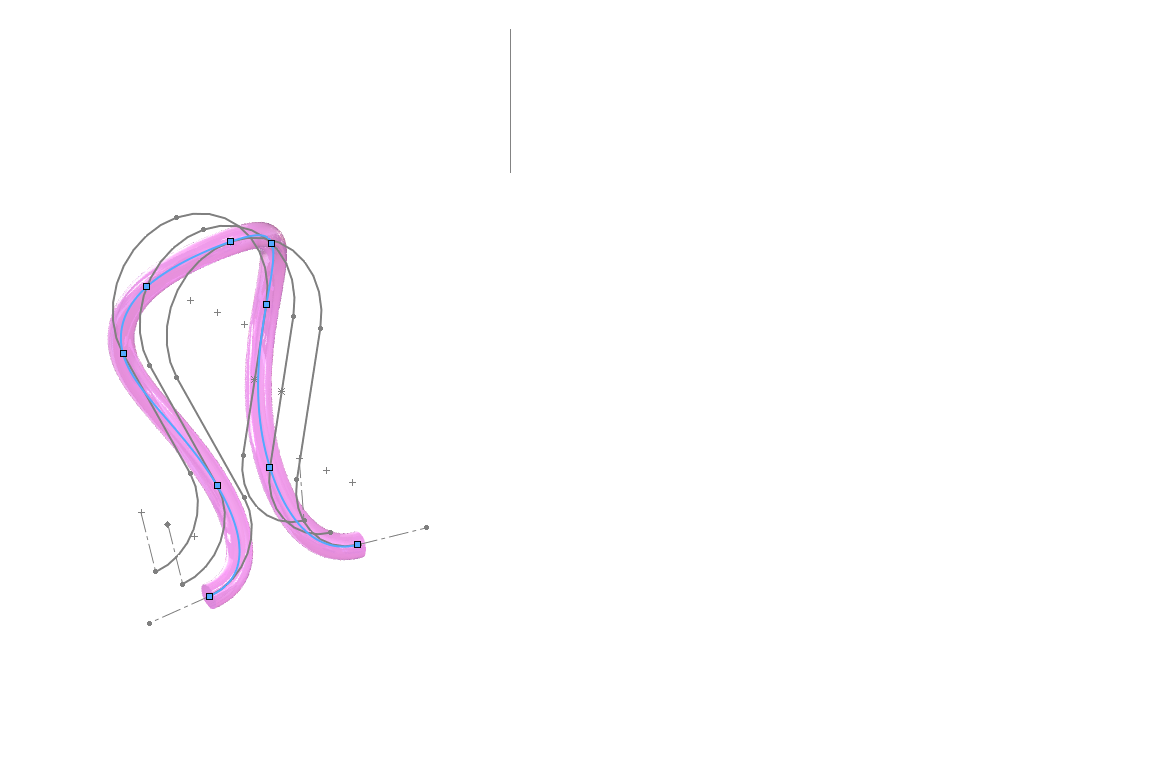


d1

d2


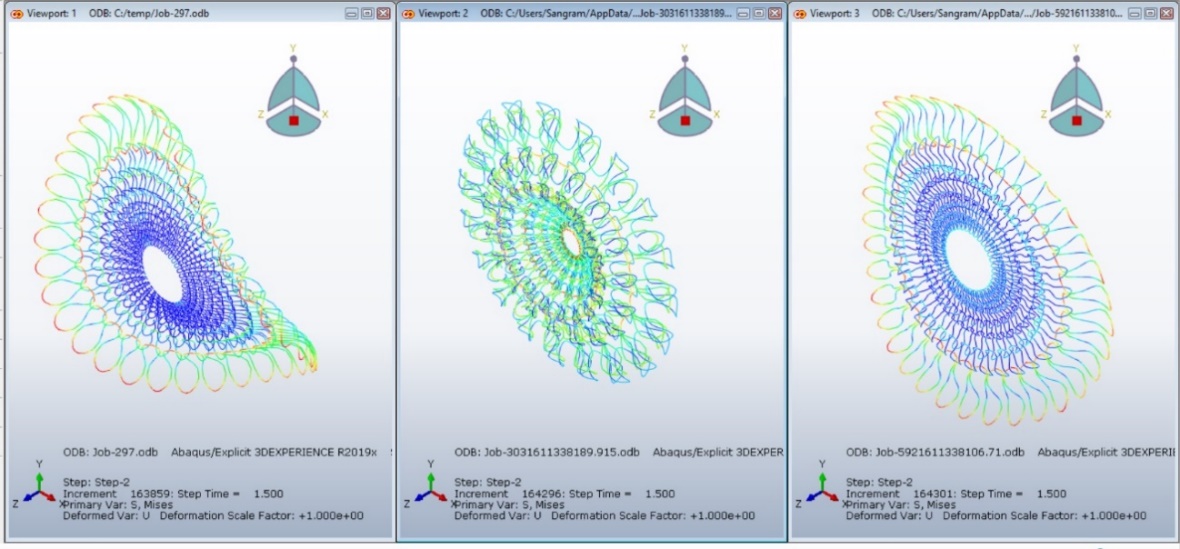

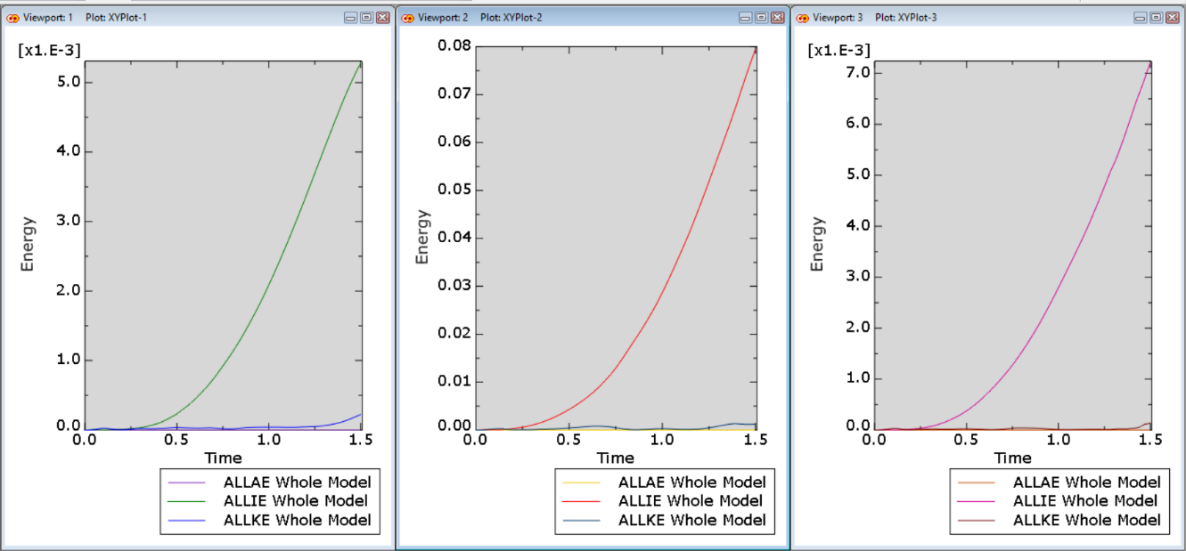


Supplementary Figure 7. Morphed Plain & Rib Knit with mass scaling (Top), Corresponding energy plot confirming that effect of mass scaling is negligible (Bottom). See ^4^ for details.

Material color coding


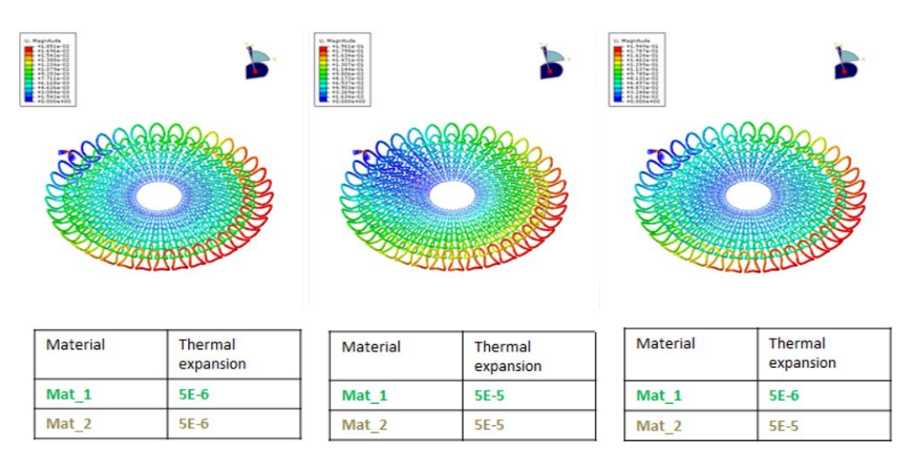

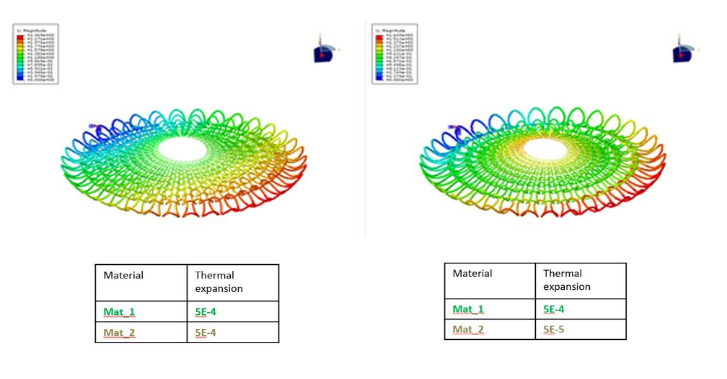

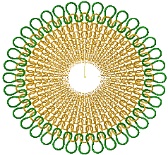


Supplementary Figure 8 Other material combinations

**References**

1. Taffetani, M., Box, F., Neveu, A. & Vella, D. Limitations of curvature-induced rigidity: How a curved strip buckles under gravity. *EPL* **127**, 14001 (2019).

2. Beauty and the Bikes: Vinyl Wrapping Motorcycles - YouTube. https://www.youtube.com/watch?v=m0qOOcIDGuk.

3. Poincloux, S., Adda-Bedia, M. & Lechenault, F. Geometry and Elasticity of a Knitted Fabric. *Phys. Rev. X* **8**, (2018).

4. ِABAQUS. Abaqus 6.14 Documentation. *Abaqus 6.14 Anal. User’s Guid.* 14 (2014).
